# The Practical Medicine Series of Year Books

**Published:** 1907-04

**Authors:** 


**The Practical Medicine Series of Year Books.** Ten volumes. Issued under the general editorial charge of **Gustavus P. Head,** M.D., Professor of Laryngology and Rhinology in the Chicago Post-Graduate Medical School. Vol VI. General Medicine. Edited by **Frank Billings,** M.D., and **J. H. Salisbury,** M.D. Chicago: The Year Book Publishers. 1906. (Price: $1.25; entire series $10.00).

The authors' names are sufficient guaranty that the subjects covered in this number of the year book series are presented sensibly and scientifically and that only the important points in the year's progress are offered. The volume contains articles on typhoid fever, malaria, yellow fever, relapsing fever, Malta fever, beriberi, dysentery, diseases of the mouth, stomach and intestines, pancreas and peritoneum; abdominal pain, epigastralgia, and tuberculosis of the thoracic duct. The article on syphilis of the liver is of especial interest for the reason that the known symptoms are clearly set forth, and every possible aid to diagnosis of this most baffling condition is given. N. W. W.

